# Minocycline Treatment Reduces Mass and Force Output From Fast-Twitch Mouse Muscles and Inhibits Myosin Production in C2C12 Myotubes

**DOI:** 10.3389/fphys.2021.696039

**Published:** 2021-07-05

**Authors:** Leonit Kiriaev, Ben D. Perry, David A. Mahns, Peter J. Shortland, Asma Redwan, John W. Morley, Stewart I. Head

**Affiliations:** ^1^School of Medicine, Western Sydney University, Sydney, NSW, Australia; ^2^School of Science, Western Sydney University, Sydney, NSW, Australia

**Keywords:** minocycline, skeletal muscle function, C2C12 myotubes, protein production, muscle force

## Abstract

Minocycline, a tetracycline-class of antibiotic, has been tested with mixed effectiveness on neuromuscular disorders such as amyotrophic lateral sclerosis, autoimmune neuritis and muscular dystrophy. The independent effect of minocycline on skeletal muscle force production and signalling remain poorly understood. Our aim here is to investigate the effects of minocycline on muscle mass, force production, myosin heavy chain abundance and protein synthesis. Mice were injected with minocycline (40 mg/kg i.p.) daily for 5 days and sacrificed at day six. Fast-twitch EDL, TA muscles and slow-twitch soleus muscles were dissected out, the TA muscle was snap-frozen and the remaining muscles were attached to force transducer whilst maintained in an organ bath. In C2C12 myotubes, minocycline was applied to the media at a final concentration of 10 μg/mL for 48 h. In minocycline treated mice absolute maximal force was lower in fast-twitch EDL while in slow-twitch soleus there was an increase in the time to peak and relaxation of the twitch. There was no effect of minocycline treatment on the other contractile parameters measured in isolated fast- and slow-twitch muscles. In C2C12 cultured cells, minocycline treatment significantly reduced both myosin heavy chain content and protein synthesis without visible changes to myotube morphology. In the TA muscle there was no significant changes in myosin heavy chain content. These results indicate that high dose minocycline treatment can cause a reduction in maximal isometric force production and mass in fast-twitch EDL and impair protein synthesis during myogenesis in C2C12 cultured cells. These findings have important implications for future studies investigating the efficacy of minocycline treatment in neuromuscular or other muscle-atrophy inducing conditions.

## Introduction

Minocycline is part of the tetracycline family of broad-spectrum antibiotics. In addition to the well-established antibacterial clinical uses of minocycline, it has gained interest as a possible treatment for neuromuscular disorders, in part because of its improved lipid solubility and uptake into cerebrospinal fluid compared to other tetracyclines (Richards et al., 1981; Orsucci et al., 2009). The mechanisms of tetracycline action in neural and neuromuscular disorders have typically been focused on its anti-apoptotic properties, particularly through mitochondria (Orsucci et al., 2009, 2012; Griffin et al., 2011). While many studies have reported the potential benefit of minocycline treatment in neuromuscular conditions such as amyotrophic lateral sclerosis (Kriz et al., 2002), neuropathies and muscular dystrophies (Orsucci et al., 2012), there is evidence that in some instances minocycline treatment may have detrimental clinical outcomes or physiological outcomes; for example, despite promising findings in animal studies, a phase III clinical trial of minocycline in ALS patients found it worsened clinical deterioration (Gordon et al., 2007). In a clinical case-study, Bokuda et al. (2012) reported skeletal muscle pigmentation, autophagic vacuoles, and scattered atrophic fibres in a 75-year-old patient who had been prescribed minocycline for several years. In *C. elegans*, minocycline treatment increased lifespan, however, it reduced protein synthesis rate (Solis et al., 2018), consistent with findings in minocycline-treated cancer cells (Jung et al., 2014).

Given the skeletal muscle atrophy which occurs in many neuropathies and neuromuscular conditions is often accompanied by sarcopenia in older populations, the independent effects of minocycline on skeletal muscle function needs to be considered. Specifically, whether there are any selective actions on different fibre types, focusing on fast-twitch fibres known to be susceptible to atrophy in sarcopenia (Deschenes, 2004; Narici and Maffulli, 2010; Picard et al., 2011; Schiaffino and Reggiani, 2011). The effect of tetracyclines, particularly minocycline, is poorly understood in reference to skeletal muscle function and atrophy. Oral minocycline treatment improved grip strength, but not muscle mass in response to cancer cachexia in mice despite reducing neural inflammation (Norden et al., 2015), and tetracycline injection did not prevent atrophy caused by external fixation, a form of limb immobilisation, despite improving expression of myogenic proteins (Shefer et al., 2008). In contrast, Obradović et al. (2016) reported hypertrophy with tetracycline treatment in cultured skeletal muscle, and in a mouse model of muscular dystrophy type 1A either minocycline or doxycycline treatment enhanced lifespan, and doxycycline treatment reduced the severity of muscle atrophy (Girgenrath et al., 2009). However, in this study they were not able to identify whether the mechanism of protection was neural or myogenic. Because of the contradictory reports in the literature investigating tetracyclines and minocycline as an anti-atrophic agent, this study aimed to investigate the effects of minocycline on skeletal muscle contractile function, size, and protein synthesis using a combination of animal and cell culture methodologies.

## Materials and Methods

### Ethics Approval

Research and animal care procedures were approved by the Western Sydney University Animal Ethics Committee A11938 in accordance with the Australian Code of Practice for the Care and Use of Animals for Scientific Purposes as laid out by the National Health and Medical Research Council of Australia. These experiments were conducted in compliance with the animal ethics checklist and ethical principles under which the journal operates.

### Animals and Treatment

Weaned (3-week-old) male C57Bl/6 mice (*n* = 10) were purchased from the Animal Resources Centre, Murdoc, WA, Australia^1^ and acclimatised for 1 week to adapt in new environment prior to start injection. Mice were housed (maximum of 3 mice per cage) in ventilated GM500 cages (Tecniplast, Buguggiate, VA, Italy) in the local animal care facility (School of Medicine, Western Sydney University). Standard rodent pellet chow (Gordon’s Specialty Stockfeeds, Yanderra, NSW, Australia) and water were available ad libitum. Basic enrichments such as nesting crinkle material and polyvinyl chloride pipe tube were provided. Mice were maintained in a temperature controlled environment throughout the entire period as previously described (Sen et al., 2019). At 4 weeks of age, mice (*n* = 5) were injected with 40 mg/kg Minocycline (Sigma-Aldrich, St Lucia, MO, United States dissolved in distilled water) intraperitoneally (29G syringe, erumo, Elkton, MD, United States) each day for five days at a similar time (10–11 am). No placebo or saline injections were used for control mice (*n* = 5). Mice were weighed every day to monitor general health. No abnormality in body condition, posture and injury were found following injections. On the 6th day mice were overdosed using intraperitoneal sodium pentabarbitone injection (250 mg/kg, Lethobarb^TM^, Tory laboratories, Glendenning, NSW, Australia).

### Muscle Preparation

The fast-twitch Tibialis anterior (TA), fast-twitch Extensor digitorum longus (EDL) and slow-twitch Soleus muscles were dissected from the hind limb. TA muscles were immediately snap-frozen while EDL and soleus muscles were tied by the tendons from one end to a dual force transducer/linear tissue puller (300 Muscle Lever; Aurora Scientific Instruments, ON, Canada) and secured to a base at the other end using 6-0 silk sutures (Pearsalls, Somerset, United Kingdom). During preparation and contractile protocols, the muscles were kept in a dissection/organ bath containing Krebs solution with composition (in mM): 4.75 KCl, 118 NaCl, 1.18 KH_2_PO_4_, 1.18 MgSO_4_, 24.8 NaHCO_3_, 2.5 CaCl_2_ and 10 glucose, 0.1% fetal calf serum, 1 drop antifoam A (BioChemika, Sigma-Aldrich) and bubbled continuously with carbogen to maintain pH at 7.4. As experiments were performed on one setup, the fast-twitch EDL muscle was mounted first whilst the slow-twitch soleus is pinned out on a dissection dish and submerged in bubbling Krebs solution.

The muscle was stimulated by delivering a current between two parallel platinum electrodes, using an electrical stimulator (701C stimulator; Aurora Scientific Instruments). All contractile procedures were designed, measured, and analysed using the 615A Dynamic Muscle Control and Analysis software (DMC version 5.417 and DMA version 5.201; Aurora Scientific Instruments). At the start of the experiment, the muscle was set to optimal length (Lo), which will produce maximal twitch force, and measured. These experiments were conducted blinded to treatment, contractile protocols and dissection were at a room temperature of ∼20–22°C.

### Initial Maximum Force

The initial supramaximal stimulus was given at 1 ms, 125 Hz for 1 s, and force produced was recorded as P_o_, the maximum force output of the muscle at L_o_.

### Force Frequency Curve and Twitch Kinetics

Force-frequency curves were generated before fatigue and after recovery to measure muscle contractile function. Trains of stimuli given at 1 ms at different frequencies, including 2, 15, 25, 37.5, 50, 75, 100, and 125 Hz for 1 s for fast-twitch EDL and 2 s for slow-twitch soleus and the force produced was measured. A 30-s rest occurred between each frequency. At 2 Hz stimulation frequency (where individual twitches are observed) the following twitch kinetics parameters were collected: twitch force, half relaxation time (HRT) and time to peak (TTP).

A sigmoid curve relating the muscle force (P) to the stimulation frequency (f) was fitted by linear regression to this data.

The curve had the equation:

$$P=P_{m\,i\,n}+\frac{P_{m\,a\,x}-P_{m\,i\,n}}{1+{\left(\frac{K^{f}}{f}\right)}^{h}}$$

From the fitted parameters of the curve, the following contractile properties were obtained: force developed at minimal (P_min_) and maximal (P_max_) stimulation at the conclusion of the force-frequency curve. Half-frequency (K_f_) is the frequency at which the force developed is halfway between (P_min_) and (P_max_), and Hill coefficient (h) which provides a way to quantify the calcium binding affinity of the muscle during contraction. These were used for population statistics.

### Fatigue and Recovery

To test the rate of fatigue development, trains of contraction stimuli were given at 1 ms, 125 Hz for 1 s for fast-twitch EDL and 1 ms, 100 Hz for 2 s for slow-twitch soleus. For fatigue development a total of 15 maximal isometric contractions were given with 1 s break after each stimuli, upon completion muscle recovery was measured by delivering the same stimuli at 30 s, 1, 3, 5, 7, 9, 11, 13, and 15 min respectively. Force values were expressed as a percentage of first fatigue contraction.

### Length Mass and Physiological CSA

Whole muscle length was measured using vernier muscle calipers while sitting at optimal length within the organ bath. All muscles were blotted dry (Whatmans filter paper DE81 grade) and weighed using an analytical balance (GR Series analytical electronic balance) following contractile procedures. Forces were normalised with respect to an estimate of physiological cross-sectional area, according to the equation CSA = MM/(Lo^∗^D) (Hayes and Williams, 1998), where MM is the muscle mass, Lo is the optimal length, and D is the density of skeletal muscle (1.06 g/cm^3^), to enable comparisons between muscles of differing sizes and weights.

### Cell Culture and Treatment

C2C12 myoblasts, a mouse derived skeletal muscle cell culture, were obtained from American Type Culture Collection (Manassas, VA, United States) and grown in Dulbecco’s Modified Eagle Medium (DMEM; Sigma-Aldrich, St. Louis, MO, United States) containing 4.5 g/L glucose and supplemented with 10% fetal bovine serum (Bovogen Biologicals, Melbourne, VIC, Australia) and antibiotics (1% of final media volume consisting of 100 U/mL penicillin and 100 μg/mL streptomycin; Sigma-Aldrich). At 90–95% confluence, cells were induced to differentiate into myotubes by replacing the growth media with DMEM containing 4.5 g/L glucose plus 2% horse serum (Sigma-Aldrich) and antibiotics for 3 days before initiating experimental treatments. Minocycline (Sigma-Aldrich), was diluted in DMEM at a final concentration of 10 μg/ml before being added to the treatment media for 48 h to observe direct effects. This concentration is similar to other studies investigating the effects of tetracycline in cultured skeletal muscle (Obradović et al., 2016).

### Tissue Sample Preparation and Western Blot Analysis

Whole cell lysates were prepared using a RIPA buffer (Sigma-Aldrich), sonicated, and cleared of cellular debris by centrifugation at 14,000*g* for 15 min. TA muscles were snap-frozen in liquid nitrogen and homogenised with RIPA buffer (Sigma-Aldrich) using a DUALL Tissue Homogeniser (Kimble Kontes). Tissue was centrifuged at 3,000*g* for 10 min, and then 14,000*g* for 15 min prior to measuring concentrations of protein in the lysates using the Bradford Protein Assay (Thermo Fisher Scientific, Waltham, MA, United States). Western blot analyses were performed as described previously (Gao et al., 2008) using nitrocellulose membranes (Biorad; Hercules, CA, United States) and commercial antibodies to Myosin (Merck, Darmstadt, Germany). Equal protein loading and electroblot transfer were verified by staining the membrane post transfer with Ponceau S Red (Sigma-Aldrich). This method has been routinely used (Perry et al., 2018) and validated as a reliable measure of protein loading with immunoblotting (Romero-Calvo et al., 2010). Following application of Chemiluminescent Substrate (SuperSignal^TM^ West Pico PLUS, Thermo Fisher), densities of detected protein bands were recorded with a BioRad chemiluminescence imager (ChemiDoc XRS+, BioRad) and densities determined using Image J (NIH, Bethesda, MD, United States).

### Protein Synthesis Assay (SUnSET)

Protein synthesis was measured using the SUrface SEnsing of Translation (SUnSET) method which measures the incorporation of puromycin into nascent peptide chains (Schmidt et al., 2009; Goodman et al., 2011). Puromycin dihydrochloride (Sigma-Aldrich) was added to the cell treatment media (1 μM final concentration) for 30 min prior to cell lysis with a standard RIPA buffer. Twenty micrograms of protein were separated in a 10% polyacylamide gel until the dye-front was ∼2 cm from the bottom of the gel. Proteins were transferred to nitrocellulose membranes (BioRad) and subsequently incubated in TBST buffer containing 5% skim milk powder and incubated overnight with a monoclonal puromycin antibody (clone 12D10, EMD Millipore, Temecula, CA, United States, diluted 1:5000 in TBST). Membranes were then incubated for 1 h in TBST containing 5% skim milk plus a secondary mouse antibody (Sigma-Aldrich, 1:10000). The total lane density was analysed using Image J (NIH).

### Statistical Analyses

Data was presented as means and standard deviation (±SD). For all data from whole mouse muscle, measurements were analysed via student unpaired *t*-tests when comparisons were made between two groups. Unpaired *t* tests were used when comparisons were made between conditions with protein synthesis and myosin protein abundance in the C2C12 myotubes. Results were considered statistically significant at *p* < 0.05, but data where the *p*-value was under <0.15 were presented in text with 95% confidence intervals for greater statistical insight and clarity into the results. All statistical tests and curve fitting were performed using a statistical software package Prism 7 (GraphPad, CA, United States).

## Results

### Muscle Length, Mass and Physiological CSA

In the fast-twitch EDL muscle, minocycline treatment reduced the mass of the muscle by 8%, and although not statistically significant (MD −1,09, 95% CI [−2.417, 0.2372], *P* = 0.102), the distribution of the 95% confidence intervals and proximity to statistical significance strongly suggests the EDL mass was lowered with minocycline treatment, while in the slow-twitch soleus muscle the mass was unaffected by minocycline treatment (Table 1). In both the fast-twitch EDL and slow-twitch soleus, there were no significant differences in muscle length between control and minocycline-treated mice. When physiological cross-sectional area was calculated from these two parameters, a similar, but not significant reduction in PCSA can be seen in minocycline treated EDL muscle (MD −0.089, 95% CI [−0.2143, 0.03608], *P* = 0.13). No significant differences in physiological cross-sectional area was seen in soleus muscle.

**TABLE 1:** Statistical analyses and sample size for muscle mass, length and physiological cross-sectional area (PCSA) of fast-twitch EDL and slow-twitch soleus muscles for control and minocycline treated groups.

	EDL			Soleus		
	Control	Minocycline	*P*-value	Control	Minocycline	*P*-value
Muscle mass (mg)	14.1 ± 1.0	13.0 ± 1.7	0.102	11.2 ± 0.7	11.5 ± 2.3	NS
Muscle length (mm)	13.2 ± 0.3	13.2 ± 0.3	NS	11.5 ± 0	11.5 ± 0	NS
PCSA (mm^2^)	1.01 ± 0.07	0.93 ± 0.1	0.13	0.92 ± 0.06	0.94 ± 0.2	NS
Sample size (muscles)	10	10		10	10	

*Values are means ± SD. NS: not significant via unpaired *t*-test.*

### Twitch Kinetics and Force

The twitch characteristics of the fast-twitch EDL and slow-twitch soleus muscles, including time to peak, half relaxation time and twitch force (absolute and specific force corrected to muscle CSA) are shown in Table 2. In the fast-twitch EDL, none of the recorded twitch characteristics were significantly different in the minocycline treated group compared to controls. In the slow-twitch soleus, both the time to reach peak twitch force (MD 10.11, 95% CI [1.338, 18.88], *P* = 0.026) and half relaxation time (MD 26.63, 95% CI [1.822, 51.43], *P* = 0.0369) were significantly increased by minocycline treatment. Absolute and specific twitch force were not statistically different between control and minocycline-treated mice (Table 2).

**TABLE 2:** Statistical analyses and sample size of single twitch kinetics for fast-twitch EDL and slow-twitch soleus muscles for control and minocycline treated groups.

	EDL			Soleus		
	Control	Minocycline	*P*-value	Control	Minocycline	*P*-value
Time to peak (ms)	29.4 ± 2.7	30.6 ± 2.7	NS	59.5 ± 8.9	69.6 ± 9.2*	0.026
Half relaxation time (ms)	14.6 ± 3.2	14.9 ± 2.3	NS	90.6 ± 18.6	117.3 ± 31.6*	0.037
Twitch absolute force (mN)	60.0 ± 7.2	60.4 ± 8.6	NS	31.5 ± 10.3	36.2 ± 10.6	NS
Twitch specific force (mN/mm^2^)	59.8 ± 9.0	66.3 ± 9.7	NS	34.4 ± 11.1	39.5 ± 13.1	NS
Sample size (muscles)	7	9		10	9	

Values are means ± SD. *P < 0.05. NS: not significant via unpaired *t*-test.

### Force Frequency Curves and Maximum Force

**FIGURE 1:**
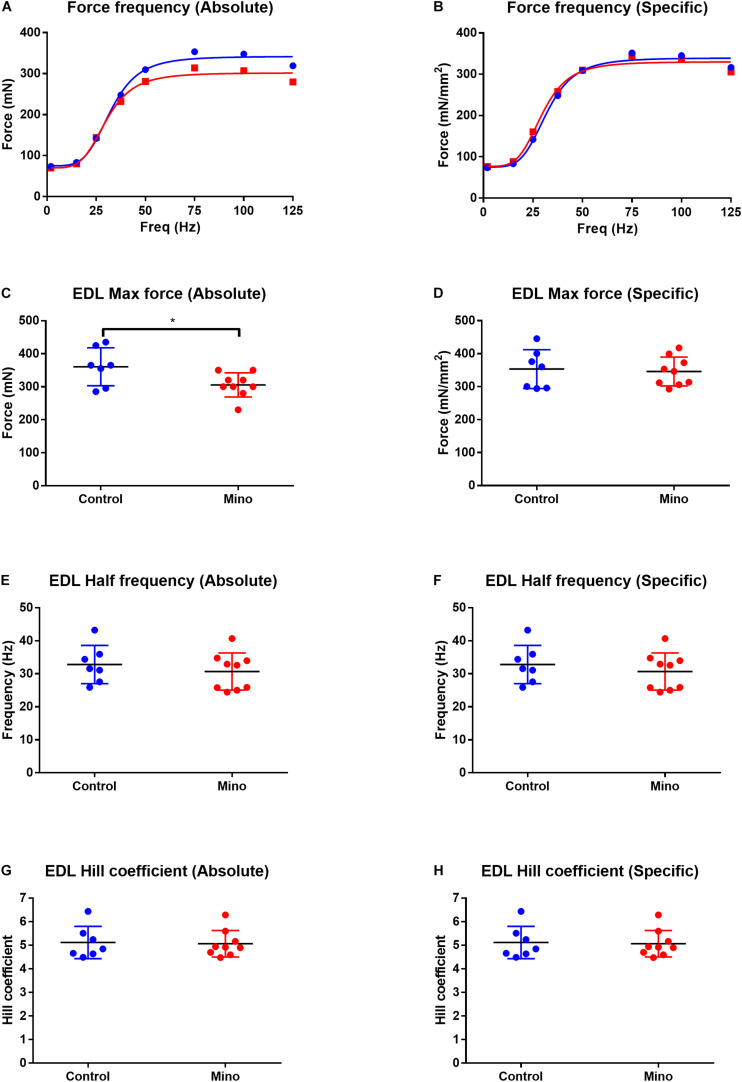
Force frequency characteristics of the fast-twitch EDL muscle expressed in absolute and specific (relative to muscle CSA) units from control (blue) and minocycline (Mino; red) groups. **(A,B)** Force frequency curve from the EDL in absolute **(A)** and specific **(B)** units, which were not different with minocycline treatment. **(C,D)** Lower EDL absolute maximal force in minocycline treated-mice **(C)**, but not when expressed relative to muscle CSA **(D)**. **(E,F)** EDL half frequency was not different with minocycline treatment in either absolute **(E)** or specific **(F)** units. **(G,H)** EDL Hill coefficient was not different with minocycline treatment in either absolute **(G)** or specific **(H)** units. *n* = 7 for Control, and *n* = 9 for Mino for all measures. *p < 0.05 via unpaired *t*-test. Values are mean ± SD.

**FIGURE 2:**
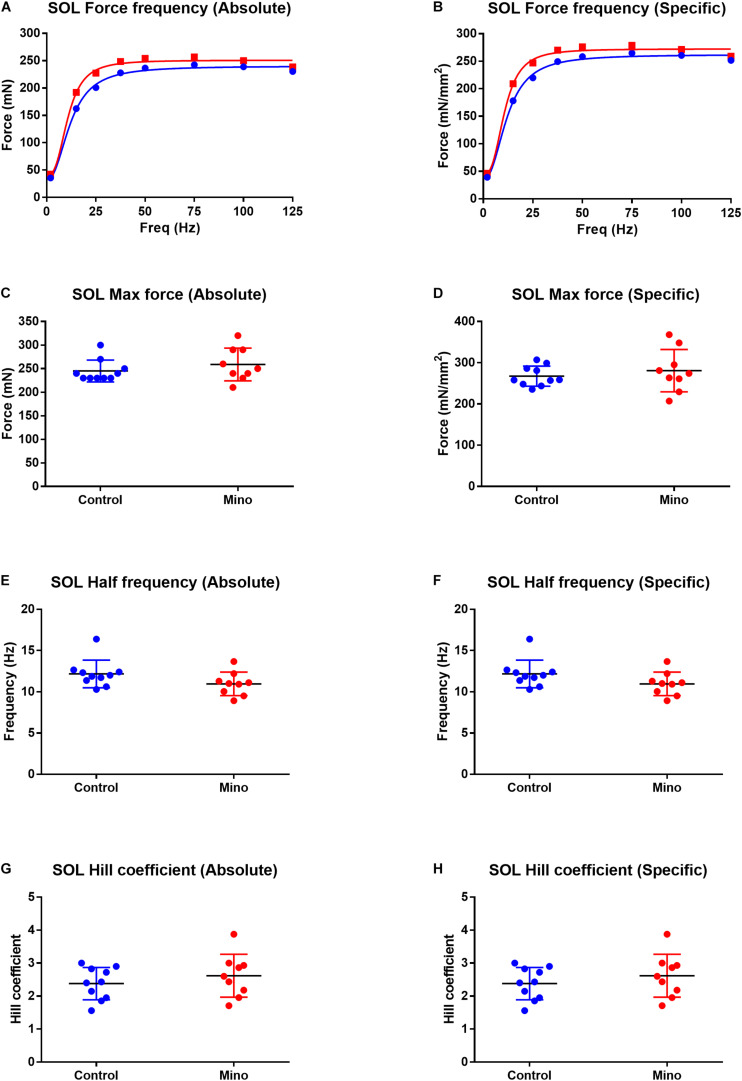
Force frequency characteristics of the slow-twitch soleus muscle expressed in absolute and specific (relative to muscle CSA) units from control (blue) and minocycline (Mino; red) groups. **(A,B)** Force frequency curve from the soleus in absolute **(A)** and specific **(B)** units, which were not different with minocycline treatment. **(C,D)** soleus maximal force was not different with minocycline treatment either in absolute **(C)** or specific **(D)** units. **(E,F)** Soleus half frequency was not different with minocycline treatment in either absolute **(E)** or specific **(F)** units. **(G,H)** Soleus Hill coefficient was not different with minocycline treatment in either absolute **(G)** or specific **(H)** units. For all measurements, *n* = 10 for Control, and *n* = 9 for Mino. Values are mean ± SD.

Fast-twitch EDL muscle treated with minocycline produced 12% less maximum force (MD −55.16, 95% CI [−105.7, −4.632], *P* = 0.035) compared to untreated controls (Figures 1A,C). All other force frequency parameters measured in EDL muscles were not altered by minocycline treatment (Figures 1B,D–H). In the slow-twitch soleus there were no statistical differences in either absolute or specific maximum force, half frequency or Hill coefficient between the control and minocycline-treated mice (Figure 2).

### Muscle Fatigability

**FIGURE 3:**
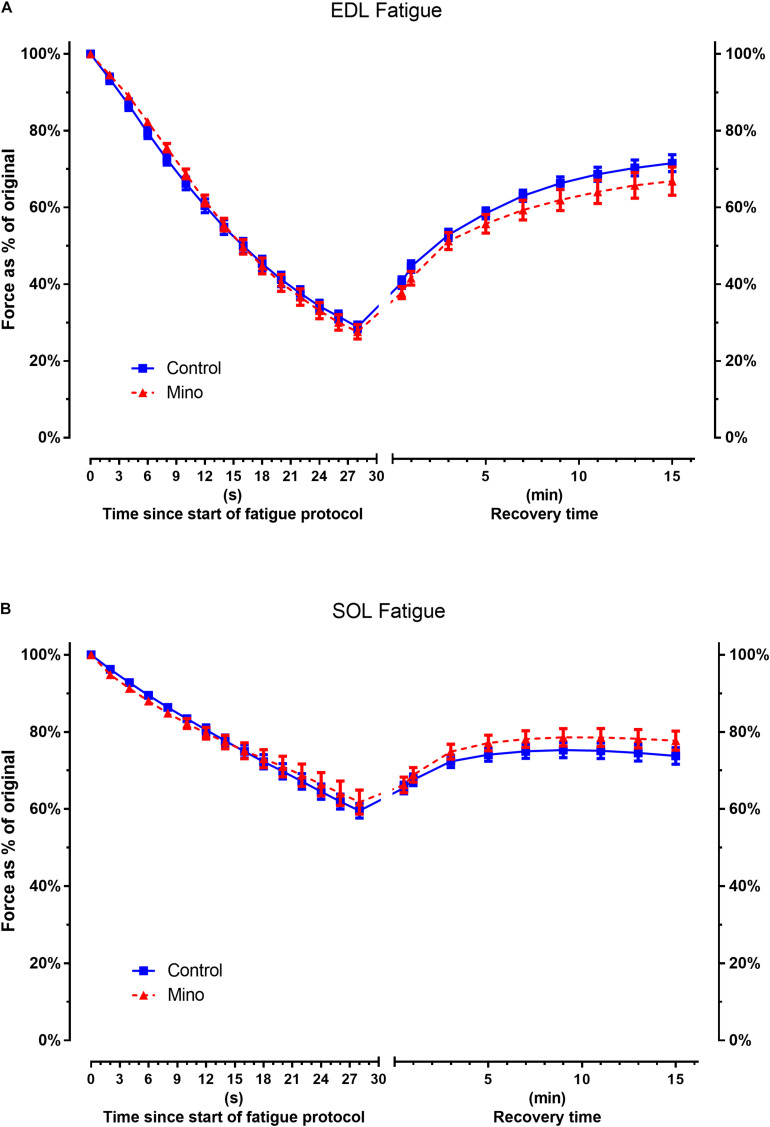
Minocycline treatment does not alter fast-twitch EDL or slow-twitch soleus fatigability. **(A)** Fast-twitch EDL force decline during fatiguing stimulated contractions or force recovery is not altered with minocycline treatment. **(B)** Slow-twitch soleus force decline during fatiguing stimulated contractions or recovery is not altered with minocycline treatment. Statistical analysis was performed at fatigue end point *t* = 30 s and recovery end point *t* = 15 min (Table 3). EDL muscle: *n* = 7 for Control, *n* = 9 for Mino. Soleus: *n* = 10 for Control, *n* = 9 for Mino. Values are mean ± SD.

The decline in force during the fatiguing protocol and force recovery were not significantly different between the control and minocycline conditions in either the fast-twitch EDL (Figure 3A) or slow-twitch soleus (Figure 3B) muscles.

**TABLE 3:** Table of descriptive statistical analysis summary for whole muscle parameters measured for A: EDL and B: Soleus.

A	EDL			
Parameter	Difference between means	95% CI	*P*-value	*N* (Control, Mino)
Muscle mass (mg)	−1.09	[−2.417, 0.2372]	0.102	(*n* = 10, *n* = 10)
Muscle length (mm)	0	[−0.2426, 0.2426]	NS	(*n* = 10, *n* = 10)
Max force (mN)	−55.16	[−105.7, −4.632]	0.035	(*n* = 7, *n* = 9)
Specific force (mN/mm^2^)	−7.264	[−62.29, 47.76]	NS	(*n* = 7, *n* = 9)
Half frequency (Hz)	−2.128	[−8.287, 4.032]	NS	(*n* = 7, *n* = 9)
Hill coefficient (%)	−0.0524	[−0.7213, 0.6166]	NS	(*n* = 7, *n* = 9)
Fatigue endpoint (%)	−4.69	[−14.58, 5.2]	NS	(*n* = 7, *n* = 9)
Recovery endpoint (%)	−1.236	[−6.53, 4.058]	NS	(*n* = 7, *n* = 9)
Time to peak (ms)	1.183	[−1.7929, 4.094]	NS	(*n* = 7, *n* = 9)
Half relaxation time (ms)	0.246	[−2.689, 3.181]	NS	(*n* = 7, *n* = 9)
Twitch absolute force (mN)	0.4314	[−8.272, 9.135]	NS	(*n* = 7, *n* = 9)
Twitch specific force (mN/mm^2^)	6.492	[−3.674, 16.66]	NS	(*n* = 7, *n* = 9)

**B**	**Soleus**			
**Parameter**	**Difference between means**	**95% CI**	***P*-value**	***N* (Control, Mino)**

Muscle mass (mg)	0.1	[−1.478, 1.678]	NS	(*n* = 10, *n* = 10)
Muscle length (mm)	−	−	NS	(*n* = 10, *n* = 10)
Max force (mN)	13.89	[−14.46. 42.24]	NS	(*n* = 10, *n* = 9)
Specific force (mN/mm^2^)	13.35	[−24.9, 51.6]	NS	(*n* = 10, *n* = 9)
Half frequency (Hz)	−1.205	[−2.715, 0.305]	NS	(*n* = 10, *n* = 9)
Hill coefficient (%)	0.237	[−0.3161, 0.7907]	NS	(*n* = 10, *n* = 9)
Fatigue endpoint (%)	2.244	[−5.317, 9.805]	NS	(*n* = 10, *n* = 9)
Recovery endpoint (%)	3.942	[−2.931, 10.82	NS	(*n* = 10, *n* = 9)
Time to peak (ms)	10.11*	[1.338, 18.88]	0.026	(*n* = 10, *n* = 9)
Half relaxation time (ms)	26.63*	[1.822. 51.43]	0.037	(*n* = 10, *n* = 9)
Twitch absolute force (mN)	4.704	[−5.435, 14.84]	NS	(*n* = 10, *n* = 9)
Twitch specific force (mN/mm^2^)	5.149	[−6.567, 16.86]	NS	(*n* = 10, *n* = 9)

*P < 0.05. NS: not significant via unpaired *t*-test.

### Protein Synthesis and Myosin Protein Abundance

**FIGURE 4:**
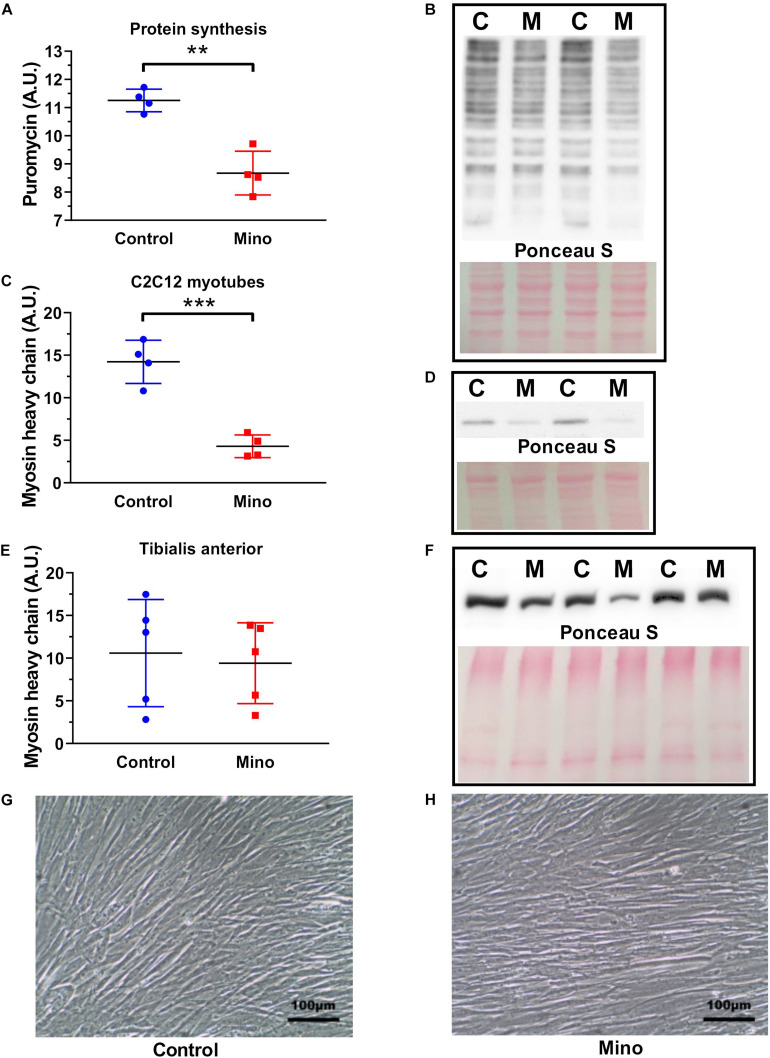
Minocycline treatment (10 μg/mL) for 48 h decreases protein synthesis and myosin heavy chain abundance in cultured C2C12 myotubes. **(A,B)** Protein synthesis was reduced by 23% with 48 h minocycline treatment (10 μg/mL), as measured via puromycin incorporation in C2C12 myotubes. **(C,D)** Myosin heavy chain abundance was reduced with 48 h minocycline treatment (10 μg/mL) in C2C12 myotubes. **(E,F)** Myosin heavy chain abundance was unchanged between minocycline treated TA muscles and untreated controls (*n* = 5 from each group). **(G,H)** Light microscope images (X20 magnification) of C2C12 myotubes treated with minocycline. Scale bars have been included in the bottom right corner of images. *n* = 4 independent experiments for each unless specified. **p < 0.01, ***p < 0.001 via unpaired *t*-tests. Values are mean ± SD.

Minocycline treatment for 48 h in cultured C2C12 myotubes caused a 23% reduction (MD −2.577, 95% CI [−3.646, −1.508], *P* = 0.0011) in protein synthesis, as measured by puromycin incorporation (Figures 4A,B). Minocycline also caused a 70% decline (MD −9.93, 95% CI [−13.44, −6.413], *P* = 0.0005) in total myosin protein abundance in myotubes (Figures 4C,D), however, a reduced effect that was not significantly different was observed in TA muscles (Figures 4E,F). Despite the decline in protein synthesis and reduction in myosin with minocycline, the cells appeared morphologically similar, and the minocycline treated cells did not show any visible signs of cell shrinkage and apoptosis (Figures 4G,H).

## Discussion

In mice, five consecutive daily treatments of minocycline intraperitoneal injection (40 mg/kg) lowered mass and absolute maximal force production in fast-twitch EDL muscle, slowed twitch rise and relaxation times in slow-twitch soleus muscle, while having no effect on muscle mass in slow-twitch soleus muscles. Furthermore, minocycline treatment in C2C12 myotubes reduced both protein synthesis and myosin heavy chain content, however, myosin heavy chain content analysis of the TA muscle showed no effect of minocycline treatment. These results demonstrate that minocycline does not directly exert any hypertrophic or functional improvements in healthy skeletal muscle. These will be important considerations for future work examining minocycline as a treatment for conditions which exhibit muscle atrophy.

This study was the first to investigate the effects of minocycline on isolated adult skeletal muscle contractile force properties, including twitch kinetics, force frequency analysis and fatigability testing. The most notable finding was the 12% decrease in absolute EDL maximal force production (Figure 1). It is noteworthy that this difference was not found when force was expressed specific to muscle physiological CSA. This finding indicates the decrease in EDL maximum force could be, in part, related to a reduction in the amount of the fast-twitch contractile protein myosin, which is reflected in the weight reduction recorded in the EDL in response to minocycline. Whilst the reduction in EDL mass was not statistically significant (<0.05), the 95% confidence interval (MD −1.09, 95% CI[−2.417, 0.2372], *P* = 0.102), combined with the ablation of the minocycline-induced decrease in max force when EDL max force was normalized to PCSA indicates that EDL mass was reduced with minocycline treatment, and is also consistent with the findings in C2C12 myotubes. In humans, and indeed in many animals, limb skeletal muscles are comprised of a mixture of fast- and slow-twitch fibres. The EDL and soleus mouse muscle are unusual in that they are predominantly comprised of fast or slow-twitch profile fibres (Augusto et al., 2004), respectively, which allows us to differentiate the fibre type response to minocycline. More specifically the mouse EDL is an mixture of approximately ∼79% type 2B (fast glycolytic), ∼16% type 2X and ∼4% type 2A (fast oxidative glycolytic) muscle fibres while the soleus contains a population of ∼39% type 2A, ∼30% type 1 (slow oxidative), ∼26% type 2X and 5% type 2B (Hettige et al., 2020). If the minocycline is reducing the amount of fast 2B/X/A myosin produced, then this would mean that the minocycline treated soleus would contain a higher proportion of slow type 1 myosin (as there was no change in mass) accounting for our finding of the slowing of the twitch and relaxation. The slower time to peak and relaxation in the soleus could be due to a shift toward a greater amount of type 1 myosin or it may be due to an increased calcium leak from the SR as a consequence of the lipid soluble minocycline entering and concentrating within the muscle fibre.

The effects of minocycline treatment on mouse-derived C2C12 myotubes were substantial. Forty-eight hours of minocycline treatment reduced protein synthesis by 23%, and substantially reduced total myosin heavy chain content (Figure 4C). This decline in protein synthesis is comparable to the degree of protein synthesis reduction found in minocycline treated *C. elegans* and in minocycline treated cancer cells (Jung et al., 2014; Solis et al., 2018). It is important to note that while the minocycline-treated cells had far lower myosin heavy chain abundance, they appeared visibly and morphologically identical under low magnification (Figures 4G,H). This indicates despite these substantial changes to myosin heavy chain content and protein synthesis, there were no overt signs of cell death. Indeed, minocycline treatment in *C. elegans* increased lifespan despite a very similar decline in protein synthesis (Solis et al., 2018), indicating despite the deleterious effects of minocycline on myosin heavy chain content and protein synthesis, minocycline may also have some undescribed protective function. However, it was interesting to note (Figures 4E,F) that in the mice minocycline treatment did not significantly alter the myosin heavy chain content of the predominately fast-twitch TA muscles.

The downregulation of myosin heavy chain content with minocycline is of importance in when considering atrophic muscle diseases and conditions. In cancer cachexia-induced muscle atrophy, there is a targeted reduction in myosin heavy chain content in both cell culture and mouse models of cancer, suggested to be linked to upregulation of ubiquitin proteasome signalling (Ladner et al., 2003; Acharyya et al., 2004) while in sarcopenia there is a preferential loss of fast-myosin (Larsson et al., 2019). Our results did not investigate the degradation pathways in response to minocycline, but given the decline in protein synthesis we report in C2C12 myotubes, it is possible that minocycline could reduce fast myosin heavy chain content through such a mechanism. In the present study, mice studied exhibited no abnormalities in body condition, posture and injury were found following 5 days of injection. Upon sacrifice and muscle removal, apart from the findings reported in this study the muscles were healthy and could sustain and recover from an extreme fatigue protocol. The C2C12 cells were monitored for 2 days post treatment, it is possible that there could be an apoptotic response post 2 days of minocycline treatment. However, given mature C2C12 myotubes cannot be treated for more than several days, it was not possible for us to properly test this possibility.

Minocycline exudes its antibacterial function via supressing protein synthesis of the prokaryotic 30S ribosome (Garrido-Mesa et al., 2013). It is apparent from our findings that minocycline can also supress protein synthesis through other mechanisms in eukaryotic cells. Jung et al. (2014) reported that minocycline reduced protein synthesis in minocycline treated cancer cells, concomitant with both a reduction in mTOR and p-P70^S6K^ and increase in eIF2α phosphorylation; if this signalling occurs in minocycline treated skeletal muscle, these are potential mechanisms for the reduced protein synthesis and myosin heavy chain content in minocycline treated C2C12 myotubes. It is also unknown whether the effects of minocycline are common to other tetracyclines, or whether the findings of our study were a unique effect of minocycline specifically. Indeed, doxycycline treatment did not elicit a reduction in myosin heavy chain content in C2C12 cultured myotubes, but instead caused hypertrophy and increased myosin heavy chain content (Obradović et al., 2016). However, Obradović et al. (2016) provided doxycycline treatment prior to myotube differentiation, suggesting doxycycline may have some positive effect on myogenesis. Conversely, another study suggested tetracycline (Sumycin) treatment impaired myotube formation in C2C12 cells (Hamai et al., 1997). Taken together, it is possible that despite being in the same broad drug classification or category, different forms of tetracyclines could elicit separate effects on skeletal muscle differentiation and morphology. Indeed, our study demonstrates that minocycline impairs protein synthesis in formed myotubes, and that the effects of tetracyclines are not limited to myotube formation and differentiation but also has a fibre-type specific impact on the adult fast-twitch EDL muscle.

In conclusion, our findings show that minocycline treatment produces mild adverse effects on absolute force production and mass in fast-twitch EDL muscle as well as a reduction of protein synthesis and myosin heavy chain content in C2C12 cultured cells. These factors should be considered in future research examining minocycline as a possible treatment for destructive and wasting muscle conditions such as Duchenne muscular dystrophy, cancer cachexia and sarcopenia.

## Data Availability Statement

The raw data supporting the conclusions of this article will be made available by the authors, without undue reservation.

## Ethics Statement

The animal study was reviewed and approved by Western Sydney University Animal Care and Ethics Committee.

## Author Contributions

LK and BP are equal first author for this publication and prepared the figures. LK, BP, AR, SH, DM, PS, and JM conceived and designed the research, and edited and revised the manuscript. LK, BP, and AR performed the experiments. LK, BP, and SH analysed the data, interpreted results of experiments, and drafted the manuscript. All authors approved the final version of manuscript and agreed to be accountable for all aspects of the work in ensuring that questions related to the accuracy or integrity of any part of the work are appropriately investigated and resolved. All persons designated as authors quality for authorship, and all those who qualify for authorship are listed.

## Conflict of Interest

The authors declare that the research was conducted in the absence of any commercial or financial relationships that could be construed as a potential conflict of interest.

## Footnotes

1 www.arc.wa.gov.au
